# A Case of Psoas Abscess

**Published:** 1870-10

**Authors:** 


### A Case of Psoas Abscess.

UNDER THE CARE OF MR. GEORGE F. ATCHLEY.

The treatment of psoas abscess is in general of a most unsatisfactory nature; for the patient usually lies upon his back for many weeks, perhaps, without much actual pain, though with great mental anxiety, the effect of long-deferred hope. When at length the abscess breaks, there is rarely any expectation of a favorable termination of the case; on the contrary, a more or less speedy death from exhaustion may be anticipated. The following short notes of a case of this kind indicate a plan—an adaptation of Prof. Lister's practice—which may reasonably be expected, Mr. Atchley thinks, to furnish a more successful result.

W. H———, aged twenty-eight, a country laborer in good condition, was admitted on the 5th of March, 1869, with a large fluctuating tumor in the upper and fore part of the left thigh, communicating, under Poupart's ligament, with another similar swelling, extending into the abdomen, along the line of the psoas muscle. The patient was unable to bend any of the lumbar and two or three last dorsal vertebræ. After resting in bed six weeks, the tumor was found to be considerably increased in size, and the contents appeared to be making their way to the surface.

The following plan was then carried out. The patient was put into a warm bath, to which was added a solution of carbolic acid sufficient to give off a very perceptible odor (about two pints of solution 1 to 30). A valvular incision was then made under the water, over the most prominent part of the swelling, and the evacuation of the contents of the abscess aided by slight pressure. As the water became opaque by the discharge of the pus, it was partially turned off, and more water and more carbolic acid added as required. Lint, saturated with the same acid and oil, was then applied to the wound, and the part bandaged, and great care taken to maintain the part under the carbolic bath throughout the operation. The wound healed in a few days. The abscess subsequently twice refilled, and a similar procedure was effected on each occasion. After the third evacuation the wound discharged small quantities of perfectly sweet matter for three weeks, and then healed permanently, and was followed by no refilling of the abscess.

The patient was discharged, well, on August 30th, just four months and a half after the first operation. He was seen a few weeks ago, when he was in full work and apparently perfect health. --*London Lancet*.

